# Diagnostic Performance of MRI Volumetry in Epilepsy Patients With Hippocampal Sclerosis Supported Through a Random Forest Automatic Classification Algorithm

**DOI:** 10.3389/fneur.2021.613967

**Published:** 2021-02-22

**Authors:** Juan Pablo Princich, Patricio Andres Donnelly-Kehoe, Alvaro Deleglise, Mariana Nahir Vallejo-Azar, Guido Orlando Pascariello, Pablo Seoane, Jose Gabriel Veron Do Santos, Santiago Collavini, Alejandro Hugo Nasimbera, Silvia Kochen

**Affiliations:** ^1^ENyS (Estudios en Neurociencias y Sistemas Complejos), Consejo Nacional de Investigaciones Científicas y Técnicas, Universidad Nacional Arturo Jauretche y Hospital El Cruce, Florencio Varela, Argentina; ^2^Hospital de Pediatría J.P Garrahan, Departamento de Neuroimágenes, Buenos Aires, Argentina; ^3^Centro Internacional Franco Argentino de Ciencias de la Información y de Sistemas (CIFASIS) - Consejo Nacional de Investigaciones Científicas y Técnicas (CONICET), Grupo de Procesamiento de Señales Multimedia - División Neuroimágenes, Universidad Nacional de Rosario, Rosario, Argentina; ^4^Instituto de Fisiología y Biofísica B. Houssay (IFIBIO), Consejo Nacional de Investigaciones Científicas y Técnicas, Departamento de Fisiología y Biofísica, Universidad de Buenos Aires, Buenos Aires, Argentina; ^5^Hospital J.M Ramos Mejía, Centro de Epilepsia, Buenos Aires, Argentina; ^6^Instituto de investigación en Electrónica, Control y Procesamiento de Señales (LEICI), Universidad Nacional de La Plata-Consejo Nacional de Investigaciones Científicas y Técnicas, La Plata, Argentina; ^7^Instituto de Ingeniería y Agronomía, Universidad Nacional Arturo Jauretche, Florencio Varela, Argentina

**Keywords:** epilepsy, volumetry, hippocampal sclerosis, random forest classifier, MRI

## Abstract

**Introduction:** Several methods offer free volumetry services for MR data that adequately quantify volume differences in the hippocampus and its subregions. These methods are frequently used to assist in clinical diagnosis of suspected hippocampal sclerosis in temporal lobe epilepsy. A strong association between severity of histopathological anomalies and hippocampal volumes was reported using MR volumetry with a higher diagnostic yield than visual examination alone. Interpretation of volumetry results is challenging due to inherent methodological differences and to the reported variability of hippocampal volume. Furthermore, normal morphometric differences are recognized in diverse populations that may need consideration. To address this concern, we highlighted procedural discrepancies including atlas definition and computation of total intracranial volume that may impact volumetry results. We aimed to quantify diagnostic performance and to propose reference values for hippocampal volume from two well-established techniques: FreeSurfer v.06 and volBrain-HIPS.

**Methods:** Volumetry measures were calculated using clinical T1 MRI from a local population of 61 healthy controls and 57 epilepsy patients with confirmed unilateral hippocampal sclerosis. We further validated the results by a state-of-the-art machine learning classification algorithm (Random Forest) computing accuracy and feature relevance to distinguish between patients and controls. This validation process was performed using the FreeSurfer dataset alone, considering morphometric values not only from the hippocampus but also from additional non-hippocampal brain regions that could be potentially relevant for group classification. Mean reference values and 95% confidence intervals were calculated for left and right hippocampi along with hippocampal asymmetry degree to test diagnostic accuracy.

**Results:** Both methods showed excellent classification performance (AUC:> 0.914) with noticeable differences in absolute (cm^3^) and normalized volumes. Hippocampal asymmetry was the most accurate discriminator from all estimates (AUC:1~0.97). Similar results were achieved in the validation test with an automatic classifier (AUC:>0.960), disclosing hippocampal structures as the most relevant features for group differentiation among other brain regions.

**Conclusion:** We calculated reference volumetry values from two commonly used methods to accurately identify patients with temporal epilepsy and hippocampal sclerosis. Validation with an automatic classifier confirmed the principal role of the hippocampus and its subregions for diagnosis.

## Introduction

Quantification of brain anatomical structures from magnetic resonance images (MR) is being increasingly used to recognize pathologic conditions such as temporal lobe epilepsy. Volumetric estimates of hippocampal size are postulated to be more sensitive than visual assessment alone, and also to improve clinical diagnosis in dementia and epilepsy (1–4).

Temporal lobe epilepsy with hippocampal sclerosis (HS) is one of the most frequent focal epilepsies in adults often refractory to pharmacological treatment; surgical resection is an effective therapeutic option for these patients achieving a seizure-free rate close to 80%.

Patients with temporal epilepsy and HS usually share clinical key features associated with the majority of seizure discharges including characteristic aura, arrest, alteration of consciousness (and amnesia), and automatisms. Relatively typical scalp EEG findings can be seen in the interictal state, at the seizure onset, during the course of the seizure, and postictally.

Hippocampal sclerosis is suspected in epilepsy patients when compatible ictal semiology and scalp EEG findings are found, but definitive diagnosis is established based on characteristic brain MR anomalies. Neuroimaging abnormalities are typically recognized in the hippocampus proper, including atrophy, loss of internal structure, and decreased T1- and increased T2-FLAIR signal intensity in clinical practice (5). Inspection of hippocampal coronal sections allows for a side-by-side comparison of asymmetry in volume, shape, and signal important for clinical diagnosis. Atrophy seems to be the most specific and signal changes the most sensitive biomarker in HS (6). Magnets with high field strengths above 3 T are able to depict subtle blurring of the internal architecture of the hippocampus on T2-weighted images (5). Originally, manual segmentation of the hippocampus based on anatomical knowledge and specific MRI landmarks was used to estimate structural volumes. Previous studies using these methods adequately identified lateralization of seizure origin in the temporal lobe of patients with HS. Earlier reports also documented a strong association between severity of histopathological anomalies and hippocampal volumes with an increased diagnostic yield of MR studies (7, 8)

The recent development of automatic volumetry methods such as FreeSurfer (FS) suite (9) and VolBrain (vB) HIPS (10), among others, makes it possible to account for hippocampal volume differences that may escape visual detection. Several studies validated the utility of hippocampal volumetry for HS detection in temporal epilepsy, mostly based on postoperative correlation or using *ex vivo* neuroimaging analysis (7–10). The potential of volumetry measures for postsurgical outcome prediction is still modest, with some improvement in reports considering subfields patterns of atrophy (11).

Since numerous publications demonstrate considerable differences in normative brain structural volumes across populations with different genetic backgrounds (12–14), volumetric estimates of the hippocampus in different populations are of particular concern. Previous reports consistently show hippocampal volume differences even when using analogous procedures (15–32). In this regard, normal anatomic variations and differences associated with the implemented methodology need to be considered for the interpretations of clinical conditions. An additional concern is that normative structural data from Latin America populations remains underrepresented.

The main objective of this work is to estimate reference values of sensitivity, specificity, and confidence intervals for classification of a local population of epilepsy patients with unilateral hippocampal sclerosis using two different volumetry approaches.

We analyzed T1 brain MRI volumetry of the hippocampus and hippocampal subfields in a cohort of 61 healthy subjects and in 57 epilepsy patients with confirmed unilateral mesial temporal sclerosis. Anatomical volumes were computed using two well-established automatic methods FS and vB. Recorded values for the hippocampus and subregions are expressed as absolute values (in cm^3^) and further normalized to brain size, quantified as a percent of total intracranial volume (TIV).

Furthermore, we provide hippocampal and subfield volume distribution for a community-based sample of healthy controls (HC) and evaluate subregion asymmetry differences in HC and between patients. We also compared the degree of asymmetry in left and right HS to investigate its relevance for diagnosis and the presence of distinctive patterns of atrophy at the subregion level.

Finally, a validation process was implemented to explore the contribution of non-hippocampal structures for group classification. This was performed using machine learning techniques, considering only FreeSurfer's morphometric information of whole-brain regions, including anatomical volumes and cortical thickness. Specifically, we used a feature selection technique to obtain the optimal number of features to discriminate between patients and HC, and then we performed three binary classifications for each group using a Monte Carlo cross-validation (MCCV) scheme (33) with a random forest classifier.

## Materials and Methods

### Participants

Patients were retrospectively enrolled based on medical records from the epilepsy unit between 2014 and 2019 at Nestor Kirchner—El Cruce Hospital at Florencio Varela, Buenos Aires, with a final diagnosis of temporal lobe epilepsy associated with unilateral right (*n* = 22, 15 females) and left (*n* = 35, 17 females) hippocampal sclerosis. Diagnosis was established using standardized practices as described in Oddo et al. (34) through clinical examination, assessment of disease history, semiology of seizures along with neuropsychological tests including prolonged video EEG, and compatible findings on 3-T MRI as suggested by ILAE (5). Thirty-one patients (54%) underwent surgical treatment with histopathology confirmation of HS after standard amygdalohippocampectomy with partial temporal lobectomy. The remaining patients are not yet operated but scheduled for surgery. Age- and sex-paired HC (*n* = 61, 44 females) were recruited mostly from local universities including students and academic personnel.

All participants gave written consent to participate and to make use of medical information for this study. The work described in this paper was carried out in accordance with the code of ethics of the world medical association (Declaration of Helsinki). Research ethics approval was obtained from the Hospital Research Ethics Board at El Cruce Hospital.

### Imaging Characteristics and Analysis Methods

Only volumetric T1-weighted images were used in this study. These images were obtained as part of the clinical protocol for epilepsy workout in our institution and were acquired using the same MR unit (Philips Achieva 3T, 8-channel head coil), as recommended on recent specialized guidelines (5). Structural images consist of a 3D T1WI (FFE) sequence, with 180 slices of 1-mm isotropic resolution, TE= 3.3 msec, TR= 2300 msec, TI= 900 msec, flip angle= 9°, and field of view (FOV)= 240 × 240 × 180. Images were exported from the scanner and transformed to Nifti format for further analysis. For the statistical analysis, the same T1 volumetric images were processed using two established and freely available methods used to calculate brain region segmentation and quantification, namely, FreeSurfer Suite v6.0 (FS) working in an offline workstation and VolBrain-Hips 2016 (vB) that provides online services running on remote servers through a website interface.

**Figure 1:**
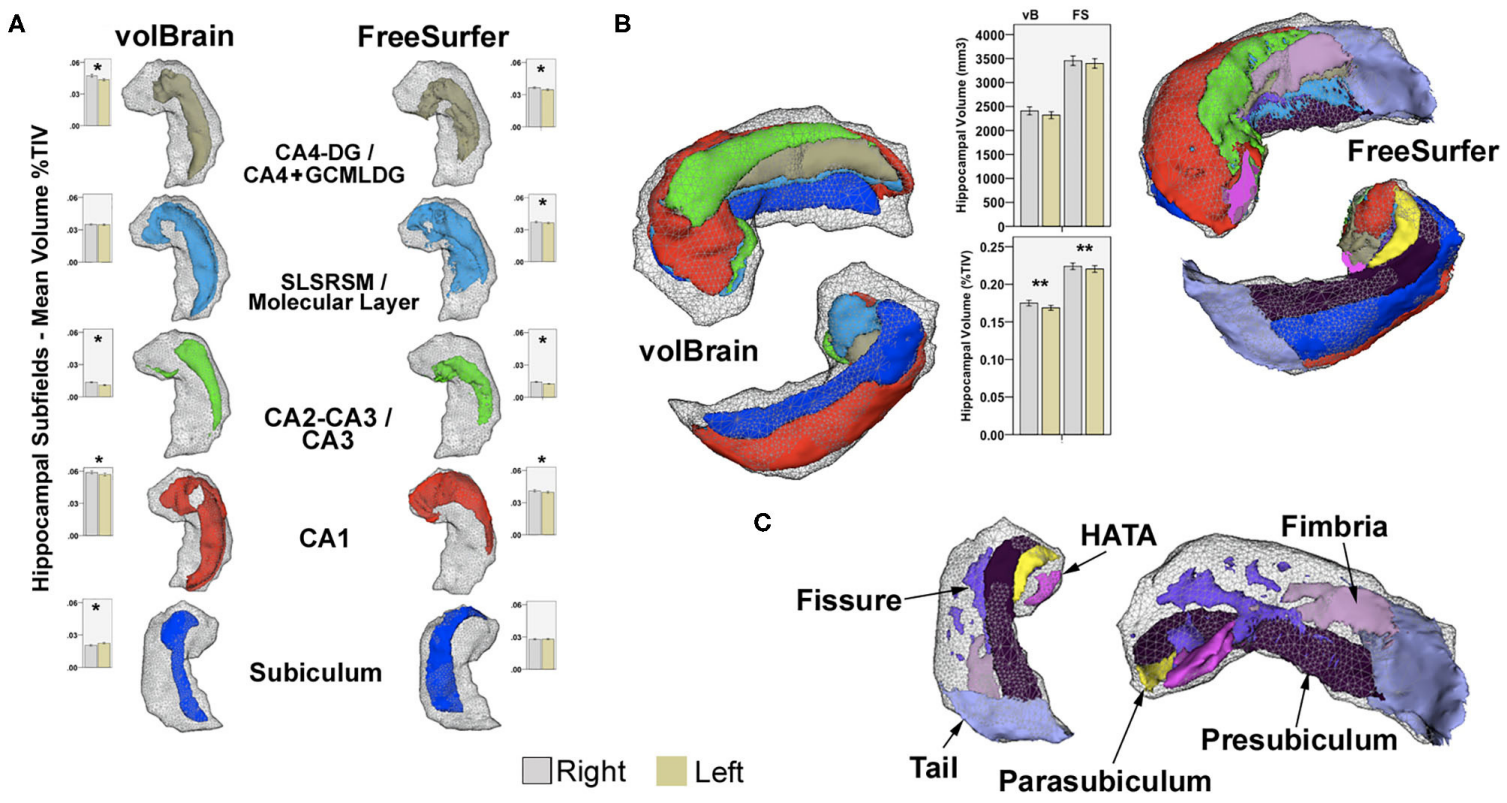
**(A)** Examples of common subfield's atlas definition using vB and FS. Boxplots represent mean volumes as percent of TIV and whiskers the 95% confidence interval for the HC group. **(B)** Right hippocampal 3D models for the same subject, constructed using all subfields from both methods in 3D Slicer^2^; boxplots represent mean hippocampal volumes for left and right hippocampi in HC expressed in mm3 and in percent of TIV. Upper models show anterior–superior view, and lower images represent inferior projections for comparison. Shaded gray-wireframe area embodies whole hippocampus representation created from standard FS segmentation; note reduced size of the vB model. Most noticeable subregion differences are related to the definition of the anterior and posterior extent of CA1 and posterior subiculum; more medially and dorsally extended in vB. Coincidentally, the hippocampal tail, pre-subiculum, and para-subiculum regions defined in FS represents at least partially overlapping areas between methods. Other deep internal hippocampal structures such as the molecular layer, GCMLDG, fissure, and fimbria are individually ascribed only in FS **(C)**. CA4-DG and CA2-CA3 subfields are jointly segmented in vB; CA4 and GCMLDF are grouped together in FS for comparison purposes. Volume differences are probably not only related to atlas definition; both approaches also show methodological discrepancies for intracranial volume computation. *Significant after Bonferroni correction. **Significant uncorrected *p* < 0.05. Paired-sample *T*-test for inter-hemispheric comparison of volumes as percent of TIV in HC.

Both methods offer validated hippocampal and hippocampal subfield segmentation through different approaches, distinct reference atlases, dissimilar processing times, and specific subfield region delineations. Output files and results from both methods were independently reviewed by two experienced neuroradiologists (JPP and GDS) looking for labeling inconsistencies and to assure quality control (no manual correction was performed). (See segmentation details for each method in Figure 1). Full documentation is available for processing details on each software platform, but here we describe a resumed version of each method.

## FreeSurfer v6.0

All T1 brain volumes were processed to obtain a complete morphometric description. Cortical reconstruction and volumetric segmentation were performed in each participant's native space on FreeSurfer's^1^ (v 6.0) image analysis suite.

Briefly, image processing included removal of non-brain tissue using a hybrid watershed/surface deformation procedure, an automatic Talairach transformation, segmentation of the subcortical WM and deep GM volumetric structures (including hippocampus, amygdala, caudate, putamen, and ventricles), intensity normalization, tessellation of the GM–WM boundary, an automatic topology correction, and surface deformation following intensity gradients to optimally place the GM/WM and GM/CSF borders at the location where the greatest shift in intensity defines the transition to the other tissue class (9).

Once the cortical models were complete, a number of deformable procedures were performed for further data processing and analysis, including surface inflation and registration to a spherical atlas—based on individual cortical folding patterns to match cortical geometry across subjects, parcellation of the cerebral cortex into units relative to gyral and sulcal structure, and creation of a variety of surface-based data—including maps of curvature and sulcal depth. These methods use both intensity and continuity information of the entire 3D MR volume from segmentation and deformation procedures to produce representations of cortical thickness, which is calculated as the closest distance from the GM/WM boundary to the GM/CSF boundary at each vertex on the tessellated surface (9). The maps were created using spatial intensity gradients across tissue classes; therefore, they were not simply reliant on absolute signal intensity. Since the ensuing maps were not restricted to the voxel resolution of the original data, they can detect submillimeter differences between groups. Procedures for the measurement of cortical thickness have been validated against histological analysis and manual measurements. FreeSurfer morphometric procedures including principal hippocampal subfields have been demonstrated to show good test–retest reliability across scanner manufacturers and across field strengths (35, 36).

The FreeSurfer v6.0 algorithm follows a generative, parametric approach which focuses on modeling the spatial distribution of the hippocampal subregions and surrounding brain structures, which is learned from labeled training data. FreeSurfer v6.0 is built with a novel atlasing algorithm and *ex vivo* MRI data from autopsy brains. The segmentation provides 15 different subregions (12 used in for this work), based on the histology and morphometry from Rosene and Van Hoesen (37) and partly also on (38–41). See Figure 1 for details on implemented atlas and segmentation.

The *ex vivo* imaging protocol yields images with high resolution and signal-to-noise ratio. The segmentation algorithm is similar to Van Leemput (42) which is appropriate for analyzing *in vivo* MRI scans of different manufacturers using different T1 contrasts.

Compared to other new methods available, FreeSurfer involves a prolonged processing time 8~24 h running on standard single-core systems but also yielding extended quantification of additional brain structures including whole-brain regions beyond hippocampal formations.

We transformed the fixed-width-column plain-text files in which were written down the FreeSurfer outputs to comma separate values (csv) plain-text files which are more suitable to be opened as a Pandas' Dataframe (Python package). To ensure that classifiers did not consider features lacking specific regional information, we eliminated general features like cortical volume, mean cortical thickness, brain volume, and ventricle volume. Finally, to avoid potential biases due to differences among the participants' head size (43), volume measures of each area were normalized as a percentage of the estimated total intracranial volume (eTIV), provided also in FreeSurfer's results.

### VolBrain—HIPS

VolBrain is a patch-based segmentation method for high-resolution hippocampus subfields. It has been validated and uses two publicly available segmentation protocols different from FreeSurfer on manually *ex vivo* segmented datasets (44, 45).

Both hippocampal segmentation protocols are available in volBrain-HIPS; Winterburn atlas disclosing 5 subregions was used for this work because it is more similar to the FreeSurfer v6.0 definition than Kulaga-Yoskovitz. VolBrain-HIPS is based on the combination of MOPAL (46), a multi-contrast extension of the OPAL (47) patch-based label fusion segmentation method and a novel neural network-based error corrector. The method uses an adaptation of MOPAL, a patch matching segmentation method to produce fast and accurate T1 brain segmentations. The method also works on standard MRI acquisition with image resolution of clinical practice as well as on single T1w or single T2w images. The VolBrain approach performs well also on mono-contrast T1w and T2w images as well as under standard resolution images that are upsampled using the LASR (48, 49) super-resolution method. The HIPS method also includes an error corrector post-processing step based on the use of a boosted ensemble of a neural network algorithm that is proposed to minimize systematic segmentation errors at post-processing. It works in a fully automated manner providing accurate results outperforming state-of-the-art methods such as MAGeT (50), ASHS (51), and SurfPatch (52) which usually require extended periods of computing time. VolBrain-HIPS takes <20 min and performs fast segmentation as well as subject-specific library registration that only requires estimating one non-linear registration over small regions to translate the whole library to the case to be segmented.

Finally, an online report is generated and results are plotted as absolute or percent values adjusted for intracranial volume against a normal reference standard for each anatomical region. Segmentation images can also be downloaded for evaluation purposes.

The same T1 volumetric images used for FreeSurfer v6.0 were uploaded to VolBrain-HIPS^3^ for this analysis, using Winterburn atlas definition for controls and patients (45). The produced final report including absolute values (mm3) and normalized to percent of brain volume were recorded for analysis.

## Supervised Classification With a Random Forest Algorithm

As suggested by several previous publications (53–59), the quantification of non-hippocampal volumes in HS patients usually shows widespread modifications, involving the thalamus, amygdala, subcortical temporal white matter, temporal pole, and entorhinal cortex among others.

To study structural changes in the brain without any bias, we used FreeSurfer v6.0 metrics, specifically parcels of cortical thickness and volumes of all the cerebral structures in combination with machine learning methods based on Random Forest Classifiers (RFC) (60). This process was based on the implementation of an automatic classification algorithm to evaluate group discrimination performance considering morphometric contribution of whole-brain structures as independent features, without any a priori consideration. The selection of RFC was based by several premises: (i) We were interested in considering linear and more importantly non-linear relationships between all the features. (ii) As the number of samples was relatively low (although it is high for this type of studies), the parameter tuning should be an optional step. (iii) The interpretability of the relevant features in the classification should be clear. Given these conditions and the experience of the research team, we selected RFC as the best suitable algorithm for the analysis (61–63).

Preprocessed features of cortical–subcortical volumes and cortical thickness normalized to estimated total intracranial volume (eTIV) were analyzed via a progressive feature elimination (PFE) procedure (64) with a Monte Carlo cross-validation scheme (33). Briefly, we performed 30 shuffle-splits in which we randomly selected 80% of the samples (with balanced classes) to train the RFC and the remaining 20% for testing to optimize the accuracy of RFC by varying the number of features from all to a single one according to its classificatory relevance. RFC quantifies a feature's importance depending on how much the average Gini impurity index decreases in the forest due to its use as a node in a tree (65). We used this score to progressively eliminate features by removing the feature with the lowest importance at each iteration. Finally, we kept the N first features in the ranking, where N is the optimal number of features such that using more than N features fails to improve the classifier's performance.

The optimal number of features was selected visually by indicating the minimal quantity at which accuracy became constant. We used this fixed number of features to compute the accuracy, the confusion matrix, and the ROC curve, and to obtain each subject's probability of being in each group (HC, left HS, and right HS).

We implemented this processing framework to perform three classifications: (i) a binary classification to discriminate HC and HS; (ii) a binary classification to discriminate left HS and right HS; and (iii) a multiclass classification to discriminate HC, left HS, and right HS. For each classification, we obtained the optimal number of features, the list of defined features, and the classification performance metrics (accuracy, confusion matrix, and ROC curves). Asymmetry metrics were not included in these analyses given the conceptual basis that RFCs consider the relationship between features, and therefore the asymmetry between hemispheres regions was indirectly taken into account.

These analyses were performed with the RFC implemented in the Python's scikit-learn package, with a fixed number of trees (2000) and the recommended number of features (P) in each split, where P is the square root of the full set of features. The maximum depth in each tree was not restricted a priori, i.e., nodes were expanded until all leaves were pure or until all leaves contained less than two samples.

### Statistical Analysis

Results were analyzed independently for each method using the Statistical Package for Social Sciences SPSS (Version 23; IBM, Armonk, New York). Volume mean average and 95% confidence intervals (CI) were calculated for each hemisphere. Receiver operating characteristic analyses were used to obtain optimal sensitivity, specificity, and 95% CI computed for left and right hippocampal sclerosis patients. A normalization of absolute values related to the total TIV was implemented and used for group comparison and correlation tests, since it was previously described as the most significant covariate to be considered (25). Normalization was performed by the following expression:

$$\begin{array}{l} \text{normalized\%}{\text{TIV}}_{\text{Subject}}=\text{AbsoluteValue}{\left(\text{cm}3\right)}_{\text{Patients}}\text{X}100/{\text{TIV}}_{\text{Subject}}. \end{array}$$

Both methods implement different atlas definitions and strategies to quantify TIV, thus precluding a direct comparison between absolute values.

Asymmetry degree was analyzed as an independent measure representing the difference between right and left regions divided by their mean (in percent) as implemented in vB and used in previous reports (25). Thus, positive values represent greater volumes on the right side.

Nominal variables were compared using the Chi square test. Paired-sample *t*-test (right vs. left) and ANCOVA (between groups) were used for normally distributed scalar variables adjusted for age and sex. Correlations were tested using the two-tailed Pearson coefficient controlling for age and sex. Significance level was adjusted for the effect of multiple comparisons using Bonferroni correction when appropriate. To test the difference between HS sides in the group of patients, an ANCOVA test was calculated on z-scores computed for each region using the following formula:

$$\begin{array}{l} \text{z}-\text{score}=\frac{\left(\text{normalized\%TI}{\text{V}}_{\text{Patients}}-\text{normalizedmean\%}{\text{TIV}}_{\text{HC}}\right)}{\text{standarddeviationmean \%TI}{\text{V}}_{\text{HC}}}. \end{array}$$

Age, sex, and clinical characteristics of epilepsy were included in the analysis as covariates.

## Results

After correction for TIV, no significant correlation was found between age and sex with hippocampal or subfield volumes (*p* > 0.05) in controls or patients.

Controls and patients were paired according to age and sex, with female prevalence (controls 44f/17m, right HS 15f/7m, and left HS 17f/18m) not reaching significant differences (p.062). Groups were not different in relation to participants' age (p. 495), control subjects with a mean of 32 (18–62y), right HS patients group with 33 (21–64y), and left HS with 34 (19–52y).

Clinical characteristics of epilepsy including seizure frequency, age at onset, and epilepsy evolution time were similar (*p* > 0.05) in both groups of patients. Right HS patients had 7 (1–30) seizures per month, disease onset at 10 (1–40y), with a duration of 23 (6–40y), and left HS epilepsy patients presented 12 (1–90), 11 (1–32y), and 22 (2–49y), respectively.

No correlation was found between clinical features of epilepsy and hippocampus or subregion volumes.

### Hippocampal Results

Estimated hippocampal volume and 95% confidence interval (CI) for controls on the right side were 3,454 cm3 (3.355–3.554)/0.2239% (0.2196–0.2283) for FS, and 2,480 cm3 (2.326–2.490)/0.1750% (0.1713–0.1787) for vB. Results for the left hippocampus were 3,398 cm3 (3.300–3.496)/0.2230% (0.2158–0.2248) for FS, and 2,320 cm3 (2.246–2.394)/0.1686% (0.1653–0.1720) for vB. Volume asymmetry was 1.6% (0.5–2.7) for FS and 3.6% (2.2–5) for vB with significant rightward lateralization (*p* < 0.003).

Mean ipsilateral hippocampal volume and 95% CI for right HS patients were 2,578 cm3 (2.401–2.755)/0.1743% (0.1603–0.1882) for FS and 1,429 cm3 (1.295–1.563)/0.1073% (0.0979–0.1167) for vB. Left hippocampus volume and 95% CI for the left HS patients were 2,560 cm3 (2.425–2.696)/0.1693% (0.1604–0.1783) for FS and 1,437 cm3 (1.324–1.549)/0.1055% (0.0981–0.1129) for vB.

Hippocampal asymmetry in the right HS group was −27.4% (−31.4/−23.5) for FS and −47% (−53.2/−41.6) for vB. Asymmetry in left HS patients was 33% (28.2/37.7) on FS and 53.2% (48–58.3) for vB. Hippocampal volumes ipsilateral to the HS side were significantly reduced compared with controls and also with the non-lesional side of right and left HS groups (p.000). Additionally, the right hippocampus was greater in left HS patients than in HC (FS, p.022) (see details in Tables 1 and 2).

**Table 1:** volBrain-HIPS results.

**Region**	**HC n:61**		**Right HS n:22**		**Left HS n:35**	
	**Mean & 95% CI volume (cm3)/ TIV-adjusted volume (%)**	**Volume asymmetry percent (%)**	**Mean & 95% CI volume (cm3)/ TIV-adjusted volume (%)**	**Volume asymmetry percent (%)**	**Mean & 95% CI volume (cm3)/ TIV-adjusted volume (%)**	**Volume asymmetry percent (%)**
Right hippocampus Left hippocampus	**2,408** (2.326–2.490) /**0.1750** (0.1713–0.1787) **2,320** (2.246–2.394) /**0.1686** (0.1653–0.1720)	**3.6** (2.2/5)**	**1,429** (1.295–1.563) /**0.1073** (0.0979–0.1167) **§** **2,293** (2.138–2.461) /**0.1727** (0.1614–0.1840)	**-47.4** (−53.2/−41.6)	**2,460** (2.331–2.590) /**0.1810** (0.1723–0.1896) **1,437** (1.324–1.549) /**0.1055** (0.0981–0.1129) Ψ	**53.2** (48/58.3)
Right CA1 Left CA1	**0.8084** (0.7772–0.8396) /**0.0587** (0.0571–0.0603) **0.7834** (0.7541–0.8127) /**0.0569** (0.0554–0.0584)	**3** (0.8/5.3)*	**0.4776** (0.4262–0.5290) /**0.0359** (0.0320–0.0398) **§** **0.8016** (0.7400–0.8631) /**0.0603** (0.0555–0.0651)	–**51.6** (−58.5/−44.8)	**0.8524** (0.8009–0.9040) /**0.0626** (0.0592–0.06602) Ψ **0.4872** (0.4454–0.5290) /**0.0357** (0.0330–0.0384) Ψ	**55.1** (49.3/60.9)
Right CA2–CA3 Left CA2–CA3	**0.1864** (0.1775–0.1953) /**0.0135** (0.0129–0.0141) **0.1504** (0.1498–0.1589) /**0.0109** (0.0103–0.0114)	**21.7** (16.8/26.7)*	**0.0924** (0.0786–0.1063) /**0.0069** (0.0059−0.0079) **§** **0.1490** (0.1318–0.1663) /**0.0112** (0.0099–0.0124)	–**47.8** (−62.5/−33)	**0.1960** (0.1803–0.2117) /**0.0143** (0.0133–0.0154) **0.0821** (0.0727–0.0916) /**0.0060** (0.0053–0.0066) Ψ	**82.2** (73.9/90.4)
Right CA4-DG Left CA4-DG	**0.6518** (0.6241–0.6796) /**0.0472** (0.0459–0.0486) **0.5996** (0.5764–0.6228) /**0.0435** (0.0424–0.0469)	**8.1** (5.7/10)*	**0.3792** (0.3334–0.4250) /**0.0284** (0.0252–0.0317) **§** **0.5999** (0.5511–0.6488) /**0.0450** (0.0418–0.0481)	–**46.3** (−55.2/−37.4)	**0.6438** (0.6066–0.6810) /**0.0473** (0.0448–0.0499) **0.3566** (0.3193–0.3940) /**0.0261** (0.0236–0.0286) Ψ	**59.1** (51.7/66.6)
Right SR-SL-SM Left SR-SL-SM	**0.4828** (0.4649–0.5006) /**0.0350** (0.0342–0.0358) **0.4780** (0.4623–0.4937) /**0.0347** (0.0339–0.0355)	**0.7** (–.9/2.5)	**0.2761** (0.2409–0.3113) /**0.0206** (0.0182–0.0230) **§** **0.4754** (0.4405–0.5104) /**0.0357** (0.0332–0.0382)	–**55** (-64.6/−45.4)	**0.5023** (0.4742–0.5303) /**0.0370** (0.0349–0.0390) **0.2819** (0.2544–0.3095) /**0.0207** (0.0188–0.0227) Ψ	**57.8** (51/54.5)
Right subiculum Left subiculum	**0.2792** (0.2686–0.2899) /**0.0203** (0.0196–0.0211) **0.3085** (0.2961–0.3201) /**0.0225** (0.0216–0.0233)	–**9.9** (−12.4/-7.4)*	**0.2042** (0.1848–0.2237) /**0.0153** (0.0140–0.0165) **§ 0.2711** (0.2515–0.2907) /**0.0204** (0.0189–0.0218) **§**	–**28.6** (−35.8/−21.3)	**0.2662** (0.2506–0.2819) /**0.0196** (0.0184–0.0207) **0.2290** (0.2143–0.2438) /**0.0168** (0.0158–0.0179) Ψ	**15.3** (9.9/20.6)

Paired-sample T-test; inter-hemispheric comparison in HC. ^*^Significant after Bonferroni correction (p < 0.001). ^**^Uncorrected (p < 0.05). Age- and sex-adjusted ANCOVA test between 3 groups; Bonferroni corrected (p < 0.05). Significant after pairwise comparisons: ^§^ Between controls and left HS. ^Ψ^ Between controls and right HS.

*Bold numbers are the mean values*.

**Table 2:** FreeSurfer v6.0 results.

**Region**	**HC n:61**		**Right HS n:22**		**Left HS n:35**	
	**Mean & 95% CI volume (cm3)/TIV-adjusted volume (%)**	**Volume asymmetry percent (%)**	**Mean & 95% CI volume (cm3)/TIV-adjusted volume (%)**	**Volume asymmetry percent (%)**	**Mean & 95% CI volume (cm3)/TIV-adjusted volume (%)**	**Volume asymmetry percent (%)**
Right hippocampus Left hippocampus	**3,454** (3.355–3.554) /**0.2239** (0.2196–0.2283) **3,398** (3.300–3.496) /**0.2203** (0.2158–0.2248)	**1.6** (0.5/2.7)**	**2,578** (2.401–2.755) /**0.1743** (0.1603–0.1882) **§** **3,386** (3204–3568) /**0.2289** (0.2134–0.2444)	–**27.4** (−31.4/−23.5)	**3,570** (3.404–3.737) /**0.2358** (0.2257–0.2459) Ψ **2,560** (2425–2696) /**0.1693** (0.1604–0.1783) Ψ	**33** (28.2/37.7)
Right CA1 Left CA1	**0.634** (0.611–0.657) /**0.0411** (0.0400–0.0421) **0.615** (0.594–0.636) /**0.0398** (0.0388–0.0408)	**3** (1.2/4.8)*	**0.483** (0.442–0.524) /**0.0326** (0.0296–0.0357) **§** **0.636** (0.587–0.685) /**0.0428** (0.0397–0.0460)	–**27.6** (−33.2/−22)	**0.680** (0.645–0.715) /**0.0449** (0.0428–0.0471) Ψ **0.465** (0.435–0.494) /**0.0307** (0.0288–0.0326) Ψ	**37.8** (33.1/42.6)
Right CA3 Left CA3	**0.217** (0.208–0.225) /**0.0140** (0.0136–0.0145) **0.190** (0.183–0.197) /**0.0123** (0.0119–0.0127)	**13.1** (10.3/16)*	**0.157** (0.144–0.171) /**0.0106** (0.0097–0.0115) **§** **0.194** (0.182–0.207) /**0.0132** (0.0121–0.0143)	–**21.6** (−28.2/−15)	**0.232** (0.218–0.246) /**0.0152** (0.0144–0.0161) Ψ **0.149** (0.139–0.159) /**0.0098** (0.0092–0.0104) Ψ	**43.4** (37.7/49.1)
Right CA4 Left CA4	**0.260** (0.252–0.268) /**0.0168** (0.0164–0.0172) **0.245** (0.237–0.252) /**0.0159** (0.0154–0.0163)	**5.9** (4/7.8)*	**0.182** (0.166–0.198) /**0.0123** (0.0111–0.0134) **§** **0.248** (0.235–0.261) /**0.0168** (0.0155–0.0181)	–**31.5** (−37.4/−25.6)	**0.272** (0.258–0.287) /**0.0180** (0.0171–0.0188) Ψ **0.174** (0.162–0.186) /**0.0115** (0.0107–0.0123) Ψ	**43.9** (37.1/50.7)
Right presubiculum Left presubiculum	**0.298** (0.288–0.307) /**0.0193** (0.0188–0.0198) **0.324** (0.313–0.335) /**0.0210** (0.0204–0.0215)	–**8.3** (−10.1/−6.5)*	**0.223** (0.207–0.239) /**0.0150** (0.0138–0.0163) **§** **0.303** (0.285–0.320) /**0.0204** (0.0191–0.0218)	–**30.6** (−36.6/−24.7)	**0.293** (0.280–0.305) /**0.0194** (0.0185–0.0203) **0.243** (0.229–0.258) /**0.0161** (0.0151–0.0171) Ψ	**19** (13.8/24.1)
Right subiculum Left subiculum	**0.429** (0.416–0.443) /**0.0278** (0.0272–0.0285) **0.432** (0.417–0.446) /**0.0280** (0.0273–0.0286)	–**0.4** (−2.1/1.2)	**0.325** (0.298–0.351) /**0.0219** (0.0200–0.0237) **§** **0.436** (0.408–0.464) /**0.0294** (0.0273–0.0316)	–**29.7** (−34.2/−25.3)	**0.434** (0.414–0.453) /**0.0286** (0.0274–0.0299) **0.333** (0.316–0.350) /**0.0220** (0.0209–0.0232) Ψ	**26.1** (21.4/30.8)
Right parasubiculum Left parasubiculum	**0.58** (0.56–0.61) /**0.0038** (0.0036–0.0039) **0.61** (0.58–0.63) /**0.0039** (0.0038–0.0041)	–**3.9** (−7.6/−0.1)	**0.47** (0.43–0.51) /**0.0032** (0.0029–0.0035) **§** **0.56** (0.52–0.61) /**0.0038** (0.0035–0.0041)	–**17.9** (−24.4/−11.4)	**0.58** (0.55–0.62) /**0.0039** (0.0036–0.0041) **0.52** (0.47–0.57) /**0.0034** (0.0031–0.0037) §Ψ	**12.4** (5.1/19.8)
Right tail Left tail	**0.539** (0.519–0.558) /**0.0349** (0.0338–0.0360) **0.543** (0.525–0.562) /**0.0353** (0.0342–0.0363)	–**0.9** (−3.4/1.4)	**0.396** (0.366–0.426) /**0.0268** (0.0244–0.0292) **§** **0.524** (0.483–0.565) /**0.0355** (0.0320–0.0390)	–**27.6** (−31.3/23.8)	**0.544** (0.513–0.575) /**0.0359** (0.0340–0.0378) **0.399** (0.376–0.423) /**0.0265** (0.0248–0.0282) Ψ	**30** (25.4/35.5)
Right fissure Left fissure	**0.148** (0.142–0.154) /**0.0096** (0.0092–0.0100) **0.140** (0.133–0.146) /**0.0090** (0.0087–0.0094)	**6** (2.6/9.3)*	**0.140** (0.126–0.154) /**0.0094** (0.0085–0.0103) **0.142** (0.128–0.155) /**0.0096** (0.0086–0.0105)	–**1.1** (−7.4/5.1)	**0.152** (0.142–0.161) /**0.0100** (0.0095–0.0105) **0.139** (0.129–0.148) /**0.0091** (0.0086–0.0097)	**8.9** (2/15.9)
Right molecular layer Left molecular layer	**0.573** (0.555–0.591) /**0.0371** (0.0363–0.0380) **0.560** (0.543–0.577) /**0.0363** (0.0355–0.0372)	**2.2** (0.9/3.5)*	**0.424** (0.392–0.456) /**0.0286** (0.0263–0.0310) **§** **0.562** (0.531–0.593) /**0.0380** (0.0354–0.0406)	–**28.4** (−33.1/−23.7)	**0.596** (0.567–0.625) /**0.0393** (0.0375–0.0411) Ψ **0.419** (0.395–0.444) /**0.0277** (0.0261–0.0293) Ψ	**34.8** (29.9/39.7)
Right GC-ML-DG Left GC-ML-DG	**0.302** (0.293–0.312) /**0.0196** (0.0191–0.0201) **0.287** (0.277–0.296) /**0.0186** (0.0181–0.0191)	**5.4** (3.7/7.1)*	**0.213** (0.195–0.231) /**0.0144** (0.0130–0.0158) **§** **0.288** (0.272–0.303) /**0.0195** (0.0180–0.0210)	–**30.6** (−35.9/−25.2)	**0.318** (0.301–0.335) /**0.0210** (0.0199–0.0220) Ψ **0.205** (0.192–0.219) /**0.0135** (0.0127–0.0144) Ψ	**43** (36.6/49.5)
Right fimbria Left fimbria	**0.81** (0.75–0.86) /**0.0052** (0.0049–0.0054) **0.81** (0.76–0.86) /**0.0052** (0.0049–0.0055)	–**0.2** (−5.2/4.6)	**0.71** (0.63–0.79) /**0.0048** (0.0041–0.0056) **0.77** (0.67–0.86) /**0.0052** (0.0045–0.0059)	–**7.2** (−15/.5)	**0.75** (0.69–0.81) /**0.0049** (0.0046–0.0053) **0.65** (0.60–0.71) /**0.0043** (0.0039–0.0047) Ψ	**14.2** (7.4/21)
Right HATA Left HATA	**0.59** (0.56–0.61) /**0.0038** (0.0036–0.0039) **0.57** (0.54–0.59) /**0.0036** (0.0035–0.0038)	**3.4** (0.3/6.5)	**0.52** (0.47–0.57) /**0.0035** (0.0031–0.0039) **§§** **0.57** (0.52–0.62) /**0.0038** (0.0035–0.0042)	–**8.4** (−17.2/.4)	**0.63** (0.60–0.66) /**0.0042** (0.0039–0.0044) Ψ **0.50** (0.47–0.53) /**0.0033** (0.0031–0.0035) ΨΨ	**23.8** (16.6/31.1)

Paired sample T-test; inter-hemispheric comparison in HC. ^*^Significant after Bonferroni correction, ^**^Uncorrected p < 0.05. Age- and sex-adjusted ANCOVA test between 3 groups; Bonferroni corrected (p < 0.05). Significant after pairwise comparisons: ^§^ Between controls and left HS; ^Ψ^ between controls and right HS. ^§§^ With left HS. ^ΨΨ^ With right HS. ^§Ψ^ Only with HC.

*Bold numbers are the mean values*.

**Figure 2:**
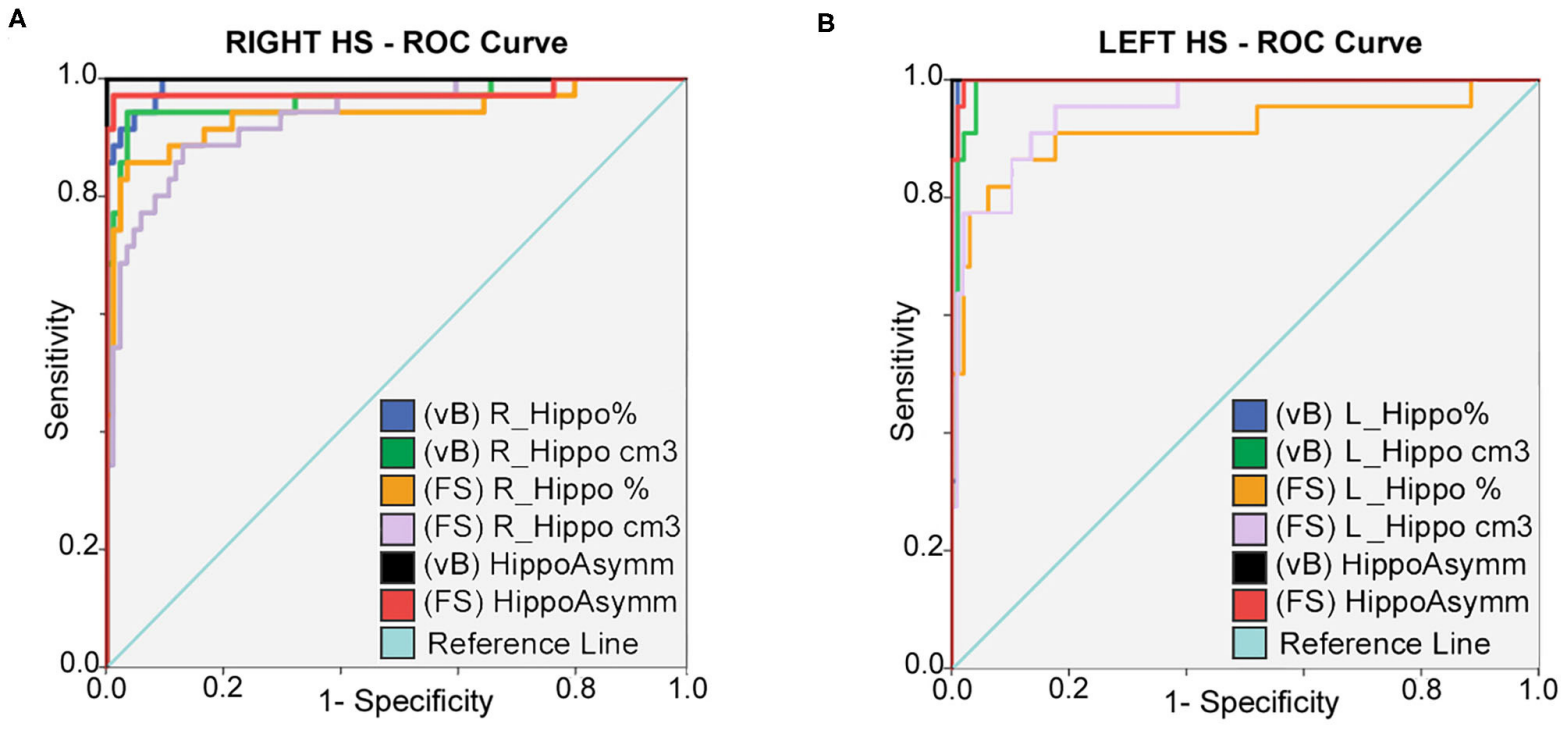
ROC curves: sensitivity and specificity for classification between HC; right **(A)** and left **(B)** HS patients. Prediction is calculated for hippocampal volumes in percent of TIV; expressed in cm3 and also using asymmetry differences for both methods. Best accuracy was obtained using asymmetry values; hippocampal volume estimates from vB showed slightly better accuracy than FS.

Hippocampal asymmetry was the most reliable indicator for accurate classification between HC and right and left HS with an AUC:1 for Vb (measured in cm3 and in brain percent), an AUC:0.998 (using cm3), and an AUC:0.977 (in brain percent) based on FS. Optimal sensitivity–specificity was also calculated using hippocampal volumes with elevated accuracy (AUC:0.914 ~ 0.993) for patient classification. Detailed results are specified in Figure 2 and Table 3.

**Table 3:** ROC curve: reference values for highest sensitivity and specificity detection for right **(A)** and left **(B)** HS; using hippocampal volumes and asymmetry values from both methods.

**Right HS**	**Ref. value**	**Sens**	**Spec-1**	**AUC**	**Sig**.
**A**					
R_Hippo (vB) %	0.1399	1.000	0.010	0.993	0.000
R_Hippo (vB) Cm3	1,910	0.909	0.031	0.989	0.000
R_Hippo (FS) %	0.1977	0.818	0.063	0.914	0.000
R_Hippo (FS) Cm3	3,044	0.864	0.104	0.952	0.000
Hippocampal (vB) Asym	−11.9	1.000	0.000	1.0	0.000
Hippocampal (FS) Asym	−8.45	1.000	0.21	0.998	0.000
**B**					
**Left HS**					
L_Hippo (vB) %	0.1442	0.943	0.048	0.992	0.000
L_Hippo (vB) Cm3	1,855	0.943	0.036	0.966	0.000
L_Hippo (FS) %	0.1920	0.857	0.036	0.937	0.000
L_Hippo (FS) Cm3	2,950	0.800	0.084	0.934	0.000
Hippocampal (vB) Asym	23.4	1.000	0.000	1.0	0.000
Hippocampal (FS) Asym	9.25	0.971	0.012	0.977	0.000

To specifically account for atrophy differences among HS sides, z-score volumes for each hippocampus were compared, and no significant differences were found (p.692, FS and p.768, vB).

### Results for Hippocampal Subfields

The mean volume and 95% CI estimates of hippocampal subfields for HC and patients are detailed in Tables 1 and 2. In the HC group, a significant rightward asymmetry of hippocampal subfields was recognized for CA1, CA2–CA3, and CA4-DG (in vB) and for CA1, CA3, CA4, molecular layer, hippocampal fissure, and GC-ML of DG (in FS). Leftward lateralization was recognized for the subiculum (vB) and pre-subiculum (FS) subregions (see details in Tables 1 and 2).

All subregions on the ipsilateral side of HS patients showed significant volume reduction compared with HC using vB, and most subfields were also reduced considering FS except for the right (p.446) and left (p.140) HATA, right and left fissure (p.1), and right ipsilateral fimbria (p.849).

Most hippocampal subfields contralateral to the sclerotic side in left HS patients revealed greater volumes compared with HC, specifically right CA1(p.048 in vB), CA1(p.002 in FS) and CA3(p.016), CA4 (p.025), HATA (p.009), molecular layer, and GC-ML-DC (p.018) on FS. Only the left subiculum (p.035 vB) of right HS patients was reduced compared with HC.

The only subregion with a significant volume difference between sides of the affected hemisphere in patients was CA2–CA3 (p.024) for the group of right HS patients (observed in vB). Accordingly, ipsilateral to the sclerotic side, CA2–CA3 (vB) and CA3 (FS) subfields in left HS patients were less atrophic than any other cornus ammonia division.

**Figure 3:**
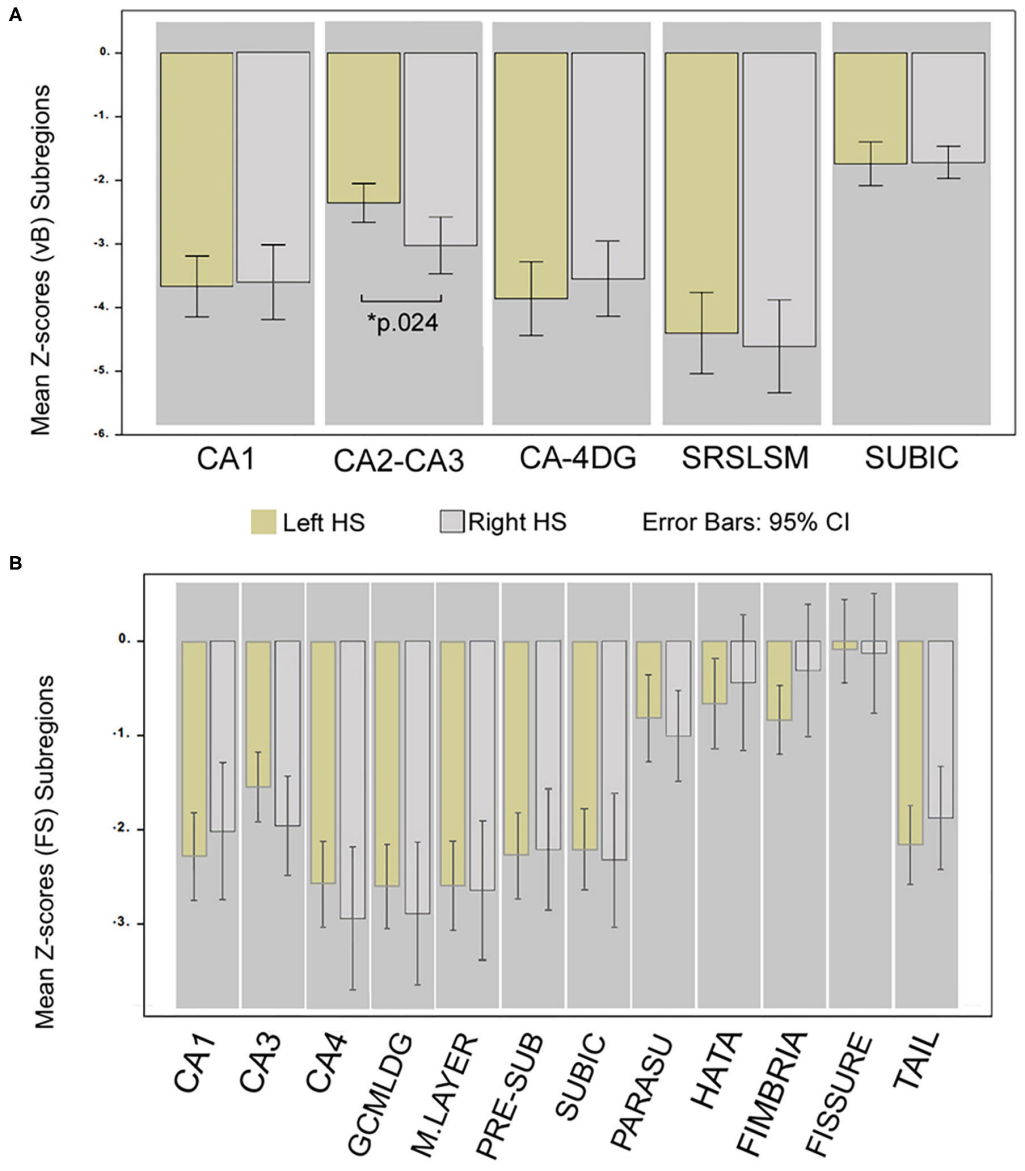
Differences in hippocampal subregion atrophy; comparison of Z-scores between HS sides. Mean Z-score volume comparison between left and right HS; obtained from vB **(A)** and from FS **(B)**. *Significant for ANCOVA test between groups; Bonferroni corrected (*p* < 0.05) adjusted for age, sex, and epilepsy characteristics. Whiskers represent 95% confidence interval.

The most atrophic subfield ipsilateral to the sclerotic side for FS were CA4, GCMLDG, and molecular layer, and SLSRSM measured in vB in both right and left HS patients. See details in Figure 3.

#### Validation With the Automatic (Random Forest) Classifier

Our supervised machine learning validation process disclosed anatomical regions that were restricted to hippocampal subregions as the most relevant features to discriminate between patients and HC. In other terms, non-hippocampal regions were not identified as relevant for the classification.

**Figure 4:**
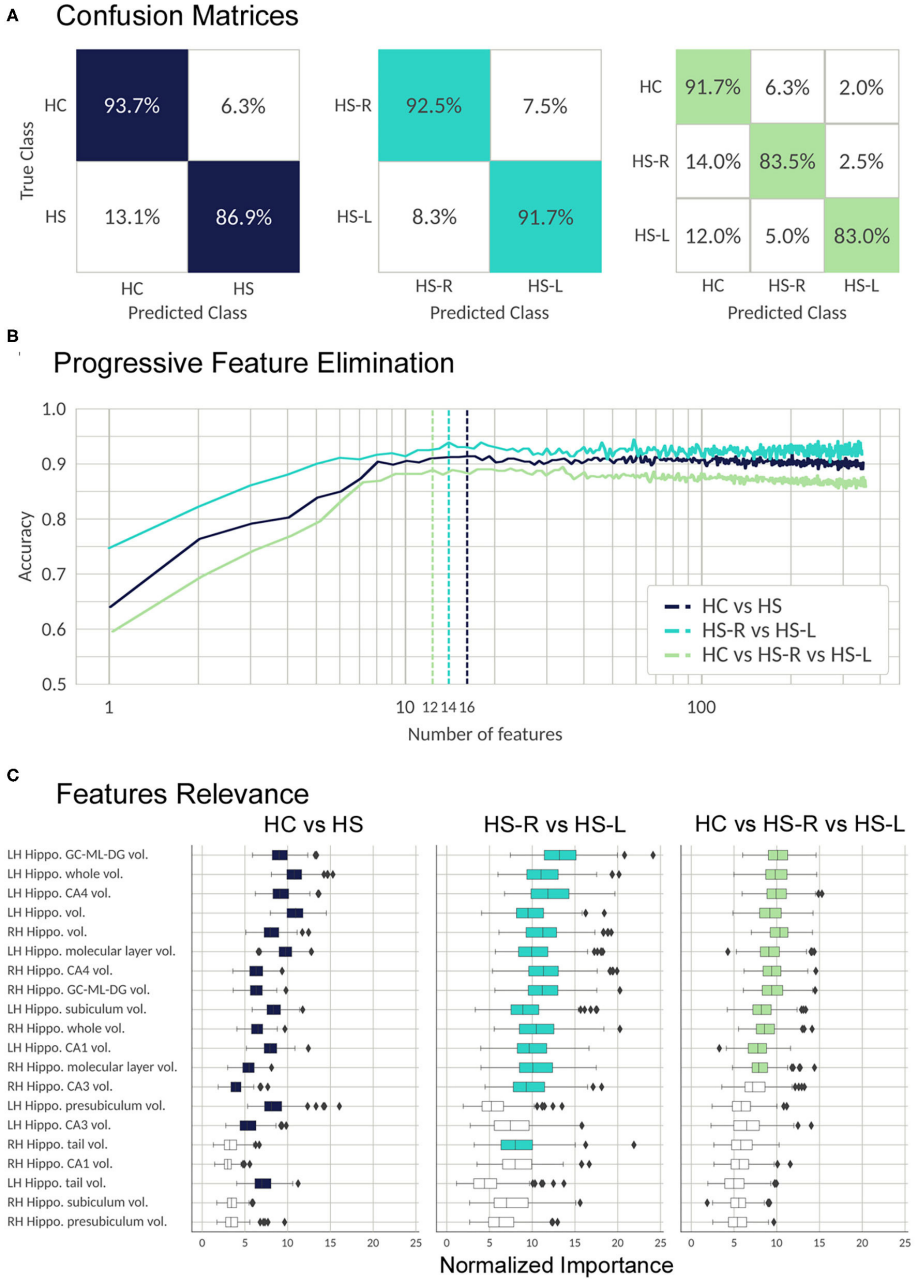
Validation process; results from the RFC algorithm. **(A)** Random forest performance. The confusion matrices show the percentage of correct (colored) and incorrect predictions for each class. The value was accumulated over the Monte Carlo cross validation (200-folds). **(B)** Progressive feature elimination. Random forest mean accuracy over the Monte Carlo cross-validation (30-folds) as a function of the number of features used to train the model. The features are sorted from most to the least important. The dashed lines show the optimal number of features for each classifier. **(C)** Feature importance for the 20 most important FS metrics. The boxplots show the normalized random forest feature importance distribution over the Monte Carlo cross-validation (200-folds). The colored boxes are the features which were selected by the progressive feature elimination procedure. The feature importance value was normalized with respect to the trivial importance level 1/N, where N is the number of features—that means, at the trivial level all the features have the same importance. Whiskers represent 95% confidence interval; small rhombuses indicate outliers.

The classifier was able to discriminate between controls and patients with a high accuracy in the three main classifications we performed: the classification between HC and patients (validation set mean accuracy: 0.907, AUC:0.960), between left and right HS patients (validation set mean accuracy: 0.91, AUC: 0.963), and between the three groups (validation set mean accuracy: 0.857, AUC: 0.960). The most important features and their relevance in each of three classifications are listed in Figure 4.

## Discussion

In this work, we define reference volumetric values and confidence intervals for hippocampus and hippocampal subfields using two commonly available approaches in a small community-based sample of healthy adults from Buenos Aires, Argentina. This is a limited sample but an important contribution to the field due to the scarce research literature on brain morphometric variations available in Latin America (66–68).

Since population variability on brain morphometric estimates are being increasingly reported (14, 69, 70, 70–72), it is important to consider the possibility of innate differences for adequate interpretation of MRI volumetry.

Several methods provide quantification of brain structures by using MRI data, including freely available softwares and online processing services that usually report adjusted values considering intracranial total volume, age, and sex as covariates. Unfortunately, wide variability exists related to the employed methodology that impairs appropriate comparisons of results between different techniques. Results are usually matched against a mixture of publicly available database of normal subjects that may not entirely account for variation among populations. Thus, absence of local references for normal and pathologic hippocampus volumes may also be a challenge for non-neuroimaging experts.

In this work, we report volumes of hippocampal structures and subregions that are specific for two different methods, evaluating patients from Latin America. The proposed reference values are intended to clarify the results obtained using two different methodologies, which are based on unequal anatomical definitions, and therefore the resulting scores cannot be directly used for cross-comparisons (see details in Figure 1).

We calculated mean volumes, confidence intervals, and cutoff estimations to recognize a regional sample of patients with confirmed unilateral mesial sclerosis and temporal lobe epilepsy with high sensitivity and specificity. Hippocampal asymmetry degree was the most accurate measure for classification regardless of the volumetry method used, as previously reported by others (3, 23, 31).

Our results are coincident with previous reports supporting rightward asymmetry for whole hippocampal volume not only in HC but also present in other animal species (73).

Interestingly, as recently reported (74), some hippocampal subregion volumes in our study were leftward lateralized in HC including the subiculum and pre-subiculum, the former based on volBrain and the latter on FreeSurfer. This discrepancy probably represents similar findings observed in overlapping areas related to known differences in atlas definitions (75) (see Figure 1).

Contrary to previous findings (31), our results did not show any significant correlation between hippocampus volume and its subfields with clinical features of epilepsy.

Few studies had focused on assessing subregion atrophy differences between HS sides based on imaging data. We found specific volume reduction of CA2–CA3(vB) in right HS patients with partial preservation in left HS patients. Future investigation using adequate methodology and involving a greater number of participants may confirm our findings. A distinctive pattern of modifications can be expected from left and right HS which are not usually considered on histopathology research, probably supporting differences in functional abilities (76–80).

To our knowledge, only one published study directly addressed asymmetry differences between hippocampal subregions among left and right HS patients using FS v6.0 (81). The authors found reduced contralateral volumes to the side of HS for presubiculum, HATA, and TAIL subfields. Unfortunately, information about known constitutional asymmetries present in HC (74) that could influence the results as in our analysis is not usually considered.

Another recent study used an approach similar to ours (but based on manual segmentation) and found greater (rather than reduced) volume of left subiculum (contrary to our findings) in right HS participants (32). Additionally, the authors also showed significant reduction of ipsilateral CA1 subfield compared against any other subregion on the sclerotic side.

An interesting observation from our analysis is a trend to find larger volumes on mesial–temporal structures contralateral to the side of HS in patients compared with HC. Diverse hippocampal subfields and also the hippocampus (FS) in the right (non-lesional) hemisphere of left HS patients support this assumption showing significant greater volumes compared to the same regions in healthy controls (Table 2). We should stress that in clinical practice the interpretation of hippocampal volumetry alone may not adequately identify some confirmed cases (~10%) with compatible clinical and paraclinical findings of HS which may only show subtle signal intensity changes on T2/FLAIR images (5, 82). Furthermore, it is important to note that a small group (~20%) of confirmed temporal lobe epilepsy patients without abnormal MRI finding will be postoperatively classified as “Gliosis only” without hippocampal sclerosis based on histopathology (83), showing no evidence of neuronal loss nor hippocampal volume reduction.

Supplementary functional imaging examinations are useful for diagnosis in temporal lobe epilepsy with HS and unremarkable MRI findings that may preserve normal hippocampal volumes. Interictal FDG-PET (2-[18F]-fluoro-2-D-deoxyglucose positron emission tomography) is a relatively widely available neuroimaging modality with high sensitivity (~80%) to disclose abnormal cortex hypo-metabolism in temporal lobe epilepsy (84, 85). Importantly, about 20% of patients with confirmed hippocampal sclerosis and normal MRI will show temporal cortex anomalies with reduced 18-FDG uptake (86, 87).

Although great progress has been made in recent years for preoperative diagnosis of HS using non-invasive methods, a considerable group of patients (20~40%) will fail to achieve complete seizure free after surgery (88, 89) following appropriate medical practices in experienced epilepsy centers. A recognized limitation of our study is the absence of histopathology information about recent standardized ILAE classification for HS subtype (ILAE HS I-III) (90) that could allow us to correlate volumetry findings with specific subfield anomalies. Nevertheless, some controversies remain concerning the role of histopathologic classification for predicting clinical evolution in HS patients and also regarding the feasibility of MRI-histopathology correlations, limited by the amount of brain sample available for examination. Additional benefits of MRI volumetry include the ability to examine the entire length of the sclerotic hippocampus and its contralateral homologous and also to consider inherited asymmetries for comparison.

Another caveat of this study is its relatively small sample size and also the uncertainty of segmentation accuracy of automated methods, to quantify structures on atrophic hippocampus. Some studies suggest that manual tracing methods may provide more accurate volumetric measurement than automated segmentation, especially in cases of HS (91, 92). However, validation results from FreeSurfer v6.0 developers indicate that subfield volumes still carry useful information, even when T1 images usually display limited contrast on the internal subregion boundaries (75). Equivalent methodology was also successfully implemented in previous studies on cognitive function and epilepsy (3, 4, 22, 23, 91, 93) with satisfactory results.

Contrary to previous observations supporting a fundamental role for cortical mesial–temporal regions, our machine learning-based validation process using an automatic algorithm failed to identify non-hippocampal structures such as the thalamus, temporal pole, fornix, or mammillary bodies as relevant for group classification. It shall be stressed that the abovementioned structures and others known to be involved in HS patients could falsely not been recognized as important due to a superior performance of hippocampal and subregion metrics in a trade-off between accuracy and number of analyzed features. Moreover, non-hippocampal anomalies preferentially involve white matter tracts (94, 95) and are usually related to prolonged epilepsy duration or high seizure frequency not considered in our validation process.

In conclusion, hippocampal anatomical structures are the most relevant features to recognize HS patients as confirmed by an automatic classification based on RFC. The local reference values proposed for hippocampal volumes and subfields may prove a useful guide for diagnosis in adult patients with temporal lobe epilepsy and suspected HS particularly for non-specialized radiologists.

Providing normal hippocampal reference values are a significant contribution to future studies focusing on regional morphometric variations in Latin America.

Finally, our results are also important for the interpretation of studies reporting hippocampal subfield volumes based on different atlas, which may show noticeable differences even when the same anatomical labels are used (96–98).

## Data Availability Statement

The raw data supporting the conclusions of this article will be made available by the authors, without undue reservation.

## Ethics Statement

The studies involving human participants were reviewed and approved by Research and Bio-ethics review board at Hospital El Cruce, Carlos N. Kirschner. The patients/participants provided their written informed consent to participate in this study. Written informed consent was obtained from the individual(s) for the publication of any potentially identifiable images or data included in this article.

## Author Contributions

JP: study design, writing, statistical analysis, and quality revision. SK: study design and manuscript edition. PS: patients selection, clinical data, and follow-up. AN: patients selection, clinical data, and demography curation and analysis. SC: statistical analysis, data pre-processing, and quality revision. AD: data processing, analysis, classification algorithm, manuscript writing, and edition. GP: classification algorithm and manuscript writing and edition. JV: data pre-processing, analysis, quality revision, and manuscript edition. MV-A: data pre-processing, analysis, quality revision, and manuscript edition. PD-K: study design, data processing, analysis, classification algorithm, and manuscript writing and edition. All authors contributed to the article and approved the submitted version.

## Conflict of Interest

The authors declare that the research was conducted in the absence of any commercial or financial relationships that could be construed as a potential conflict of interest.

## Footnotes

^1^https://surfer.nmr.mgh.harvard.edu/

^2^https://www.slicer.org

^3^https://volbrain.upv.es/
